# A248 RISK OF ARRHYTHMIA IN INFLAMMATORY BOWEL DISEASE

**DOI:** 10.1093/jcag/gwad061.248

**Published:** 2024-02-14

**Authors:** M Narous, Z Nugent, I Rabinovich-Nikitin, L Kirshenbaum, C Bernstein

**Affiliations:** University of Manitoba Max Rady College of Medicine, Winnipeg, MB, Canada; University of Manitoba Max Rady College of Medicine, Winnipeg, MB, Canada; University of Manitoba Max Rady College of Medicine, Winnipeg, MB, Canada; University of Manitoba Max Rady College of Medicine, Winnipeg, MB, Canada; University of Manitoba Max Rady College of Medicine, Winnipeg, MB, Canada

## Abstract

**Background:**

Inflammatory bowel disease (IBD) has been shown to be associated with stroke or early atherosclerosis. Atrial fibrillation (AF), being the most common arrhythmia seen clinically, can be a consequence of systemic inflammation. The association between IBD and arrhythmia has been studied in recent literature with inconclusive evidence.

**Aims:**

1. To determine the risk of arrhythmia (defined as AF, other supraventricular or ventricular tachycardia) in IBD.

2. To compare the risk of arrhythmia amongst IBD medications.

3. To assess mortality in IBD with arrythmia as compared to controls.

**Methods:**

The risk of arrhythmia was determined in a retrospective population-based cohort study using the University of Manitoba IBD Epidemiology Database (11,432 IBD cases and 109,582 matched controls). Arrhythmia risk in IBD was adjusted for presence of comorbidities of the Charlson Comorbidity Index (CCI). The effect of IBD medications on the development of arrhythmia was assessed in a nested case-control study of individuals with IBD. Follow-up started on date of IBD diagnosis until arrhythmia diagnosis, emigration out of the province of Manitoba, death, or March 31, 2018. Cases were censored at date of first database identification of a diagnosis of heart failure or myocardial infarction. All-cause mortality was a secondary outcome compared across non-IBD controls and IBD cases with arrhythmia using proportional hazards regression analysis was done.

**Results:**

Persons diagnosed with IBD were more likely than controls (HR 1.51; 95% CI 1.30-1.76) to develop arrhythmia. When controlling for CCI comorbidities, the significant association between IBD and arrhythmia remains. Medications including 5-ASA, thiopurines, and TNFa inhibitors were not associated with arrhythmia, with OR 0.99 (95% CI, 0.68-1.45), 1.06 (95% CI, 0.57-1.97), and 0.90 (95% CI, 0.31-2.61), respectively. All-cause mortality was slightly increased in persons with IBD in analyses unadjusted and adjusted for the presence of arrhythmia, with HR's of 1.13 (1.07-1.19) and 1.12 (1.06-1.18). Increased mortality was more pronounced in CD, but UC did not raise risk of mortality.

**Conclusions:**

Persons with IBD have a higher risk of arrhythmia even prior to a diagnosis with heart disease. IBD medications did not raise risk of arrhythmia in our dataset. All-cause mortality, with the development arrhythmia as a time dependent variable, was significantly raised in CD as compared to non-IBD controls, but not in UC. Further data are necessary to establish the risk of arrhythmia in IBD, especially with stratification by setting of arrhythmia diagnosis and severity of IBD based on Montreal classification.

**Funding Agencies:**

None

